# ADCY5-related dyskinesia: a case report

**DOI:** 10.1186/s42466-022-00204-w

**Published:** 2022-08-15

**Authors:** Shih-Ying Chen, Chen-Jui Ho, Yan-Ting Lu, Chih-Hsiang Lin, Meng-Han Tsai

**Affiliations:** 1 Department of Neurology, Kaohsiung Chang Gung Memorial Hospital, Niaosung District, 123 Dapi Road, Kaohsiung, 83301 Taiwan; 2 Medical School, College of Medicine, Chang Gung University, Taoyuan City, Taiwan; 3 Genomics and Proteomics Core Laboratory, Department of Medical Research, Kaohsiung Chang Gung Memorial Hospital, Kaohsiung, Taiwan

**Keywords:** ADCY5, Dyskinesia, Whole exome sequencing, Chorea, Dystonia

## Abstract

Adenylyl cyclase 5 (ADCY5) related dyskinesia is a rare disorder characterized by early-onset paroxysmal choreoathetosis, dystonia, myoclonus, or a combination of the above, which primarily involved the limbs, face, and neck. Other common clinical features are axial hypotonia and episodic exacerbation of dyskinesia. Both sporadic and inherited cases have been reported and the predomiant mode of inheritance is autosomal dominant. Herein, we describe the first ADCY5-related dyskinesia patient in Taiwan.

**Dear editor,**

Adenylyl cyclase 5 (ADCY5) related dyskinesia is a rare disorder characterized by early-onset paroxysmal choreoathetosis, dystonia, myoclonus, or a combination of the above, which primarily affects the limbs, face, and neck [1]. Other common clinical features are axial hypotonia and episodic exacerbation of dyskinesia, which may precipitate by sleep, emotional stress, intercurrent illness, or without any trigger [2]. ADCY5-related dyskinesia occurs either in a sporadic or inherited pattern. The predominant mode of inheritance is autosomal dominant, although recessive inheritance has been reported [3]. To the best of our knowledge, no Taiwanese patient has been reported, we hereby describe a patient with ADCY5-related dyskinesia.

A 24-year-old female, from a non-consanguineous family, was born as a full-term baby without any perinatal injury. At 11-month-old, she developed intermittent involuntary movements including eye blinking, mouth smacking, shoulder shrugging, and four limb choreic movement. The movement could not be suppressed by herself and persisted during sleep. Her motor milestones were delayed, she started to walk at age of two and talked at 2.5 years old after early intervention. The involuntary movement progressed slowly until her adolescence and became stationary thereafter. Episodic exacerbation during stress, menstrual period, illness, or initiation after resting was noted. Although she suffered from frequent minor injuries due to falls or dyskinetic movement, she could still take care of herself without assistance and finish her high school education.

She came to our Neurological Clinic at age of 23. Neurological examination showed slow saccade, dysarthria, oculomotor and tongue impersistent, axial hypotonia, facial dyskinesia, choreoathetosis over four limbs, and neck dystonia. The remainder of the neurological examination was normal. Her parents and older brother didn’t have similar symptoms. The basic metabolic workups were normal. Electroencephalogram showed no epileptiform discharges during dyskinesia and brain magnetic resonance imaging was unremarkable.

Whole exome sequencing revealed a known pathogenic heterozygous variant in the *ADCY5* gene (NM_183357:Exon 2:c.1252C>T:p.Arg418Trp) using the Geneyx software (Geneyx, Inc. Israel) (Fig. 1). The variant was not presented in the gnomAD (especially the East Asian population) or the TaiwanBiobank database. None of the remaining rare variants was associated with dyskinesia or chorea. The variant was confirmed de novo by Sanger sequencing of the patient and her parents (Fig. 2). The variant was classified as pathogenic by the ACMG/AMP guideline (PS3 + PM1 + PM2 + PM6 + PP3) [4].

**Fig. 1:**
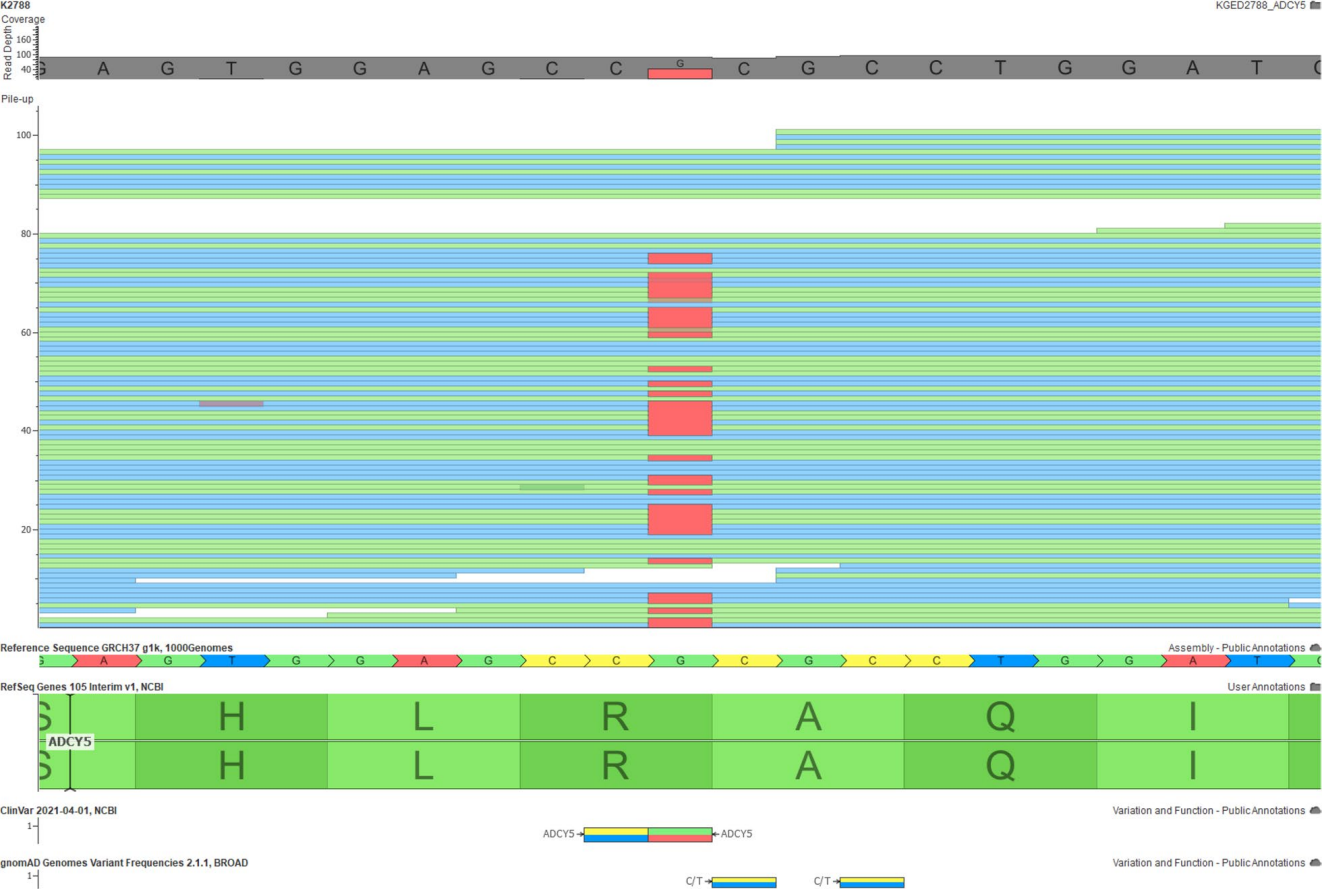
Whole-exome sequencing in the proband. The figure shows the alignment of the reads around the pathogenic variant (c.1252C>T:p.Arg418Trp) of the BAM file visualized using the GenomeBrowse software (Golden Helix, Inc. USA) (https://www.goldenhelix.com/products/GenomeBrowse/). The top panel illustrates the coverage of the reads, and the middle panel shows the actual mapping of the reads with blue indicating forward reads and green indicating reverse reads. The bottle panel shows the reference genome (CRCH37) and information from the gnomAD and ClinVar databases

**Fig. 2:**
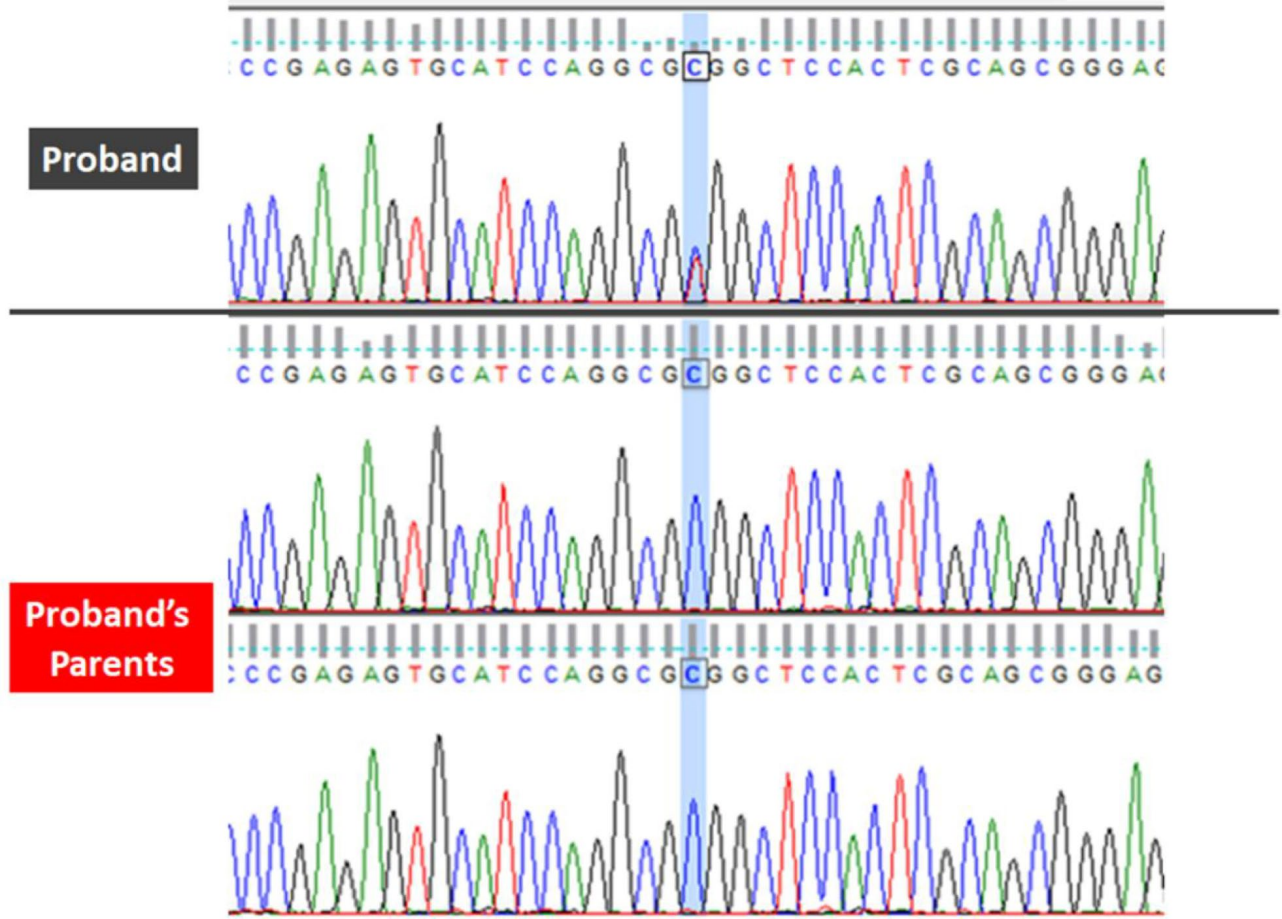
Sanger sequencing in proband and proband’s parents. Sanger sequencing of variant c.1252C>T shows a peak of both T and C allele in the proband, whereas her parents have homozygous wildtype allele C

Several medications were prescribed, including Trihexphenidyl, Levetiracetam, Zonisamide, Propranolol, Acetazolamide, Piracetam, and Alprazolam, but no clinical improvement was observed. Intriguingly, the patient reported some benefits after consumption of coffee. Therefore, caffeine tablets were prescribed and started from 100 mg in the morning and 100 mg in the noon, which showed a partial reduction of her dyskinesia. However, when we further increased the caffeine dose to 300 mg per day, she had a paradoxical worsening of dyskinesia. She is now taking 200 mg of caffeine daily.

Adenylyl cyclases are a family of enzymes that convert adenosine triphosphate (ATP) into cyclic adenosine monophosphate (cAMP). *ADCY5* is the major isoform expressed in the heart and the brain, which is highly expressed in the striatum and the nucleus accumbens. ADCY5 protein has two cytosolic domains C1 and C2, which subdivide into catalytic (C1a and C2a) and regulatory (C1b and C2b) subdomains. The catalytic subdomains interact to form an ATP-binding pocket. The pathogenic variant (p.Arg418Trp) is located in the C1a domain, where most pathogenic variants locate. Pathogenic variants were also found in other domains (C1b and C2a). It has been reported that variants in C1a and C2a domains cause a more severe phenotype than in the C1b domain [2]. Nearly all reported *ADCY5* pathogenic variants caused gain of function change, and most cases are presented with paroxysmal hyperkinesia over limbs, face, and neck, even during sleep.

The dyskinesia is burdensome to our patient, although slowly progressive and not life-threatening, but it still affects her daily activities and results in frequent injuries. In recent two case reports, a dramatic improvement was noted after caffeine consumption [5, 6]. Partially response was also observed in our patient taking 200 mg caffeine daily, but paradoxical worsening was found at higher doses. Caffeine can antagonize A2A receptors, which may counteract the abnormal ADCY5 activity [5].

Bilateral deep brain stimulation (DBS) of the globus pallidus interna is another treatment option. A significant response of dyskinesia was noticed in eight ADCY5-related dyskinesia cases in recent studies. However, other clinical features, such as axial hypotonia, did not respond to DBS [7–10]. DBS may be an option for this patient if it is covered by the national health insurance program in the future.

In summary, we reported the first ADCY5-related dyskinesia patient in Taiwan, who presented with a partial response to caffeine. The treatment response in our patient is suboptimal, further novel therapies are needed to improve the quality of life of -patients with ADCY5-related dyskinesia.

## Data Availability

All data and materials of this study are available on request from the corresponding author.

## Abbreviations

ADCY5: Adenylyl cyclase 5
DBS: Deep brain stimulation
ATP: Adenosine triphosphate
cAMP: Cyclic adenosine monophosphate
